# E2 enzyme Bruce negatively regulates Hippo signaling through POSH-mediated expanded degradation

**DOI:** 10.1038/s41419-023-06130-2

**Published:** 2023-09-12

**Authors:** Sha Song, Xianjue Ma

**Affiliations:** 1 College of Life Sciences, Zhejiang University, Hangzhou, 310058 Zhejiang China; 2 Key Laboratory of Growth Regulation and Translational Research of Zhejiang Province, School of Life Sciences, Westlake University, Hangzhou, 310024 Zhejiang China; 3 Westlake Laboratory of Life Sciences and Biomedicine, Hangzhou, 310024 Zhejiang China

**Keywords:** Cell proliferation, Cancer models, Ubiquitylation

## Abstract

The Hippo pathway is a master regulator of organ growth, stem cell renewal, and tumorigenesis, its activation is tightly controlled by various post-translational modifications, including ubiquitination. While several E3 ubiquitin ligases have been identified as regulators of Hippo pathway, the corresponding E2 ubiquitin-conjugating enzymes (E2s) remain unknown. Here, we performed a screen in *Drosophila* to identify E2s involved in regulating wing overgrowth caused by the overexpression of Crumbs (Crb) intracellular domain and identified Bruce as a critical regulator. Loss of *Bruce* downregulates Hippo target gene expression and suppresses Hippo signaling inactivation induced tissue growth. Unexpectedly, our genetic data indicate that Bruce acts upstream of Expanded (Ex) but in parallel with the canonical Hippo (Hpo) -Warts (Wts) cascade to regulate Yorkie (Yki), the downstream effector of Hippo pathway. Mechanistically, Bruce synergizes with E3 ligase POSH to regulate growth and ubiquitination-mediated Ex degradation. Moreover, we demonstrate that Bruce is required for Hippo-mediated malignant tumor progression. Altogether, our findings unveil Bruce as a crucial E2 enzyme that bridges the signal from the cell surface to regulate Hippo pathway activation in *Drosophila*.

## Introduction

Hippo signaling pathway was initially identified in *Drosophila* using genetic screens aiming to uncover genes whose disruption would cause distinct overgrowth phenotype [1–3]. Following studies using various animal models revealed evolutionarily conserved roles of Hippo pathway in regulating numerous biological activities under both physiological and pathological conditions, including cell death, cell proliferation, embryogenesis, stem cell renewal, immune surveillance, and not surprisingly, tumorigenesis [4, 5]. The core components of the *Drosophila* Hippo pathway consist of *Expended (Ex)*, *warts (wts)*, *hippo (hpo)*, *yorkie (yki)*, and *scalloped (sd)*. Ex, Merlin, and Kibra form a complex that recruits the adapter protein Salvador, which in turn recruits the core kinase Hpo [reviewed in [4]]. Hpo phosphorylates Wts, which in turn phosphorylates and inactivates Yki, once Hippo signaling is inactivated, Yki localizes to nuclei and binds to Sd to initiate transcription of target genes such us *expanded* (*ex*), *Drosophila inhibitor of apoptosis 1* (*diap1*), and *four-jointed* (*fj*). Apart from the classical kinase cascade-mediated phosphorylation, the most abundant type of post-translational modifications (PTMs) in regulating Hippo pathway is ubiquitination [6], a reversible event sequentially catalyzed by ubiquitin-activating enzymes (E1s), ubiquitin-conjugating enzymes (E2s) and ubiquitin ligases (E3s). Numerous E3s have been identified as regulators of Hippo pathway in both *Drosophila* and mammal [6–11], however, it remains unknown which E2 is essential for the regulation of Hippo pathway.

Unlike many other conventional pathways, Hippo signaling is not activated by dedicated ligand/receptor complexes. Still, several transmembrane proteins are recognized as crucial upstream inputs that can initiate Hippo signal transduction, among which *crumbs* (*crb*), the apical-basal polarity protein, is one of the most intensively studied one in *Drosophila* [12–19]. Crb contains a large extracellular domain and a short intracellular domain, the function of Crb largely depends on the intracellular domain (*crb*^*intra*^). Crb promotes apical localization of Ex through a direct interaction, and overexpression of *crb*^*intra*^ inactivates Hippo pathway and induces Yki-dependent overgrowth through ubiquitination-dependent degradation of Ex, at least in part, through E3s including *Plenty of SH3s* (*POSH*) and *supernumerary limbs* (*slmb*) [12–16, 20].

**Fig. 1:**
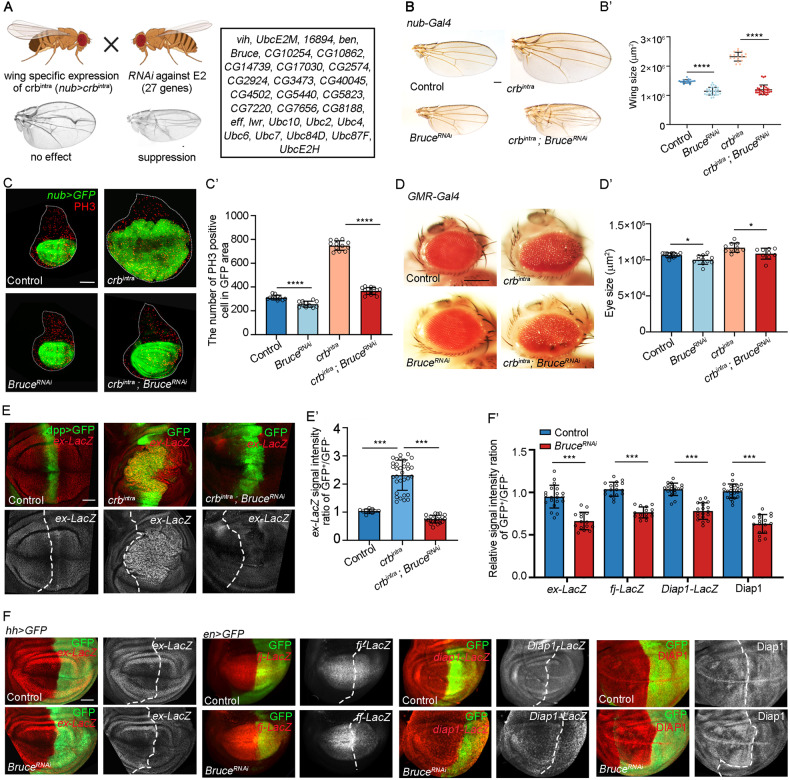
*Bruce* is required for *crb*^*intra*^-induced overgrowth and Yki target gene expression. **A** A scheme of RNAi screen strategy to identify E2s whose depletion could suppress *crb*^*intra*^-induced wing overgrowth, candidate E2 enzymes screened are listed. **B** Light micrographs of adult wings bearing indicated genotypes are shown. Quantification of wing size (B’), *n* = 19, 29, 18, 32 (from left to right). **C** GFP labeled wing discs bearing indicated genotypes. (C’) Quantification of PH3 positive cells of indicated genotypes, *n* = 12, 10, 12, 14 (from left to right). **D** Light micrographs of adult eyes bearing indicated genotypes are shown. Quantification of eye area (D’), *n* = 13, 11, 10, 10 (from left to right). **E** Wing pouch regions from larvae with indicated genotypes stained for expression of *ex-lacZ*. Quantification of relative *ex-lacZ* intensity (E’), *n* = 11, 32, 21 (from left to right). **F** Third instar wing discs expressing *Bruce*^*RNAi*^ by *hh* promoter or *en* promoter stained for expression of *ex-lacZ*, *fj-lacZ*, *diap1-lacZ*, and DIAP1. Quantification of relative staining intensity (F’), *n* = 20, 15, 17, 15, 19, 19, 23, 17 (from left to right). Mean ± SD, *p < 0.05, ***p < 0.001, ****p < 0.0001; one-way ANOVA tests (B’, C’, D’, and E’) or two-tailed Student’s *t* tests (F’). Scale bars: 200 μm for (**B**) and (**D**), 50 μm for (**F**); 20 μm for (**E**).

Here, we performed a genetic candidate screen in *Drosophila* aiming to identify E2 coding genes whose disruption could suppress wing phenotype caused by ectopically express *crb*^*intra*^ during wing development (Fig. 1A). We identified *BIR repeat containing ubiquitin-conjugating enzyme* (*Bruce*) as a crucial regulator of Hippo signaling. Unexpectedly, we found the Bruce-Ex axis acts in parallel with Hpo-Wts cascade to regulate Yki activity and showed that Bruce synergizes with POSH to regulate Ex ubiquitination and degradation. We further demonstrated that Bruce is essential for Hippo pathway-dependent malignant tumor progression.

## Results

### Bruce is required for crb^intra^-induced overgrowth and Yki target gene expression

We have previously identified the E3 ubiquitin ligase POSH as a regulator of Hippo pathway and showed that POSH is essential for overexpression of Crb^intra^-induced growth in *Drosophila* wing [15], however, the E2 ubiquitin-conjugating enzyme that modulates Hippo pathway has remained unknown. To uncover the corresponding E2s, we performed RNAi screen against *Drosophila* coding E2s that have homologs in human, aiming to find candidates whose depletion could suppress *crb*^*intra*^-induced wing overgrowth phenotype (Fig. 1A). We focused on one strong candidate named Bruce, a giant Baculovirus IAP repeat (BIR) domain containing E2 that plays essential roles in development, apoptosis, and autophagy [21–24]. Depletion of *Bruce* under the control of *nubbin* (*nub*) promoter efficiently reduces the size in both adult and developing wing and dramatically suppressed *crb*^*intra*^-induced tissue overgrowth and cell proliferation in both stages (Fig. 1B-C’). Although *Bruce* knockdown did not result in a noticeable phenotype in the eyes, it effectively mitigated the enlarged rough eye phenotype caused by overexpressing *crb*^*intra*^ in the developing eye using *GMR*-Gal4 (Fig. 1D-D’). Notably, we also observed a comparable suppression phenotype using an independent *Bruce* RNAi strain (Fig. S1), indicating that *Bruce* is essential for *crb*^*intra*^ overexpression induced overgrowth.

As Crb^intra^ overexpression regulates organ size in imaginal discs through inhibiting Hippo signaling [12–14, 16, 20], next, we asked whether Bruce plays a role in regulating Hippo target gene expression. Ectopic expression of *crb*^*intra*^ along the anterior-posterior boundary of wing disc under the control of *dpp-Gal4* strongly upregulated *ex* transcription, as monitored by the *ex-lacZ* staining (Fig. 1E-E’), whereas coexpression of *Bruce* RNAi significantly inhibited *crb*^*intra*^-driven *ex* upregulation (Fig. 1E-E’). Furthermore, we found that under physiological conditions, depletion of *Bruce* specifically in the posterior region of wing epithelium robustly downregulated the endogenous expression of multiple Yki targets, including *ex*, *fj*, and *diap1* (Fig. 1F-F’). Together, these data indicate that Bruce is required for Yki-dependent target gene expression.

### Bruce genetically acts upstream of Ex but in parallel with Hpo-Wts cascade to regulate Yki activity

**Fig. 2:**
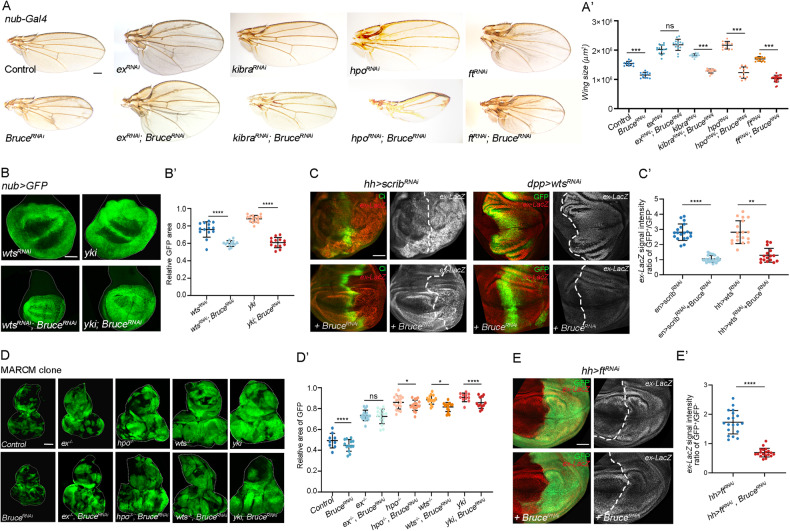
Bruce acts upstream of Ex but in parallel with Hpo-Wts cascade. **A** Light micrographs of adult wings bearing indicated genotypes are shown. Quantification of wing size (A’), *n* = 12, 12, 14, 14, 16, 18, 11, 11, 15, 23 (from left to right). **B** GFP labeled wing pouch regions with indicated genotypes are shown. Quantification of relative GFP region size (B’), *n* = 14, 13, 15, 13 (from left to right). **C** Wing pouch regions from larvae with indicated genotypes stained for expression of *ex-lacZ*. Quantification of relative *ex-lacZ* intensity (C’), *n* = 19, 22, 18, 16 (from left to right). **D** Eye-antennal discs of *ey-Flp*-MARCM-induced GFP positive mosaics clones with indicated genotypes. Quantification of clone size was shown in (D’), *n* = 9, 13, 16, 16, 18, 18 15, 15, 11, 12 (from left to right). **E** Wing pouch regions from larvae with indicated genotypes stained for expression of *ex-lacZ*. Quantification of relative *ex-lacZ* intensity (E’), *n* = 15, 14 (from left to right). Mean ± SD, n.s. not significant; *p < 0.05, **p < 0.01, ****p < 0.0001; two-tailed Student’s *t* tests (E’) or ordinary one-way ANOVA tests (A’, B’, C’, and D’). Scale bars: 200 μm for (**A**), 100 μm for (**B**, **D**); 50 μm for (**E**).

To further pinpoint where Bruce executes its function in Hippo signaling, we performed genetic epistasis analysis between Bruce and different Hippo pathway components. As previously demonstrated, inhibition of Hippo signaling by knocking down *wts*, *hpo*, *kibra*, *ex* or overexpression of *yki* under the *nubbin* (*nub*) promoter induced overgrowth phenotype in the developing wing or adult wing (Fig. 2A-B’). We found that depletion of *Bruce* significantly suppressed loss of *kibra*, *hpo*, or *wts*, and overexpression of *yki*-induced overgrowth phenotype (Fig. 2A-B’), as well as *wts-RNAi* induced *ex-lacZ* upregulation (Fig. 2C-C’), indicating that genetically Bruce might act in parallel or downstream of Yki. Similarly, the overgrowth and *ex* upregulation induced by depletion of cell polarity gene *scribble* (*scrib*), another upstream regulator of Hippo pathway, was also suppressed by knockdown of *Bruce* (Fig. 2C-C’). Although overexpression of full-length Bruce (Bruce^FL^) completely suppresses *reaper*-induced apoptotic eye phenotype [23], it has no effect on the small eye phenotype caused by Hpo overexpression (Fig. S2A, B). Furthermore, overexpression of *Bruce* did not lead to a further increase in excessive proliferation caused by the *ex* or *kibra* mutants, suggesting that Bruce overexpression alone is not sufficient to activate Yki in eye epithelium (Fig. S2C-C’). Remarkably, we noticed an unexpected genetic interaction between Ex and Bruce, unlike the rest of the Hippo components, *ex* loss-induced wing overgrowth phenotype remained unaffected upon the depletion of *Bruce* (Fig. 2A-A’). Furthermore, we confirmed these intriguing findings in *Drosophila* eye-antennal epithelium using mosaic analysis with a repressible cell marker (MARCM) based clone induction system (Fig. 2D-D’). Given that Ex could inhibit Yki activation by tethering it in the cytosol [25, 26], these data suggest that Bruce positively regulates Yki activity by acting upstream of Ex but in parallel with the Kibra-Hpo-Wts cascade.

Apart from Crb, *fat* (*ft*) represents another well-studied transmembrane regulator of the Hippo pathway, loss of *ft* resulted in Ex-dependent overgrowth and increased Yki-driven *ex* transcription (Fig. 2E-E’), but paradoxically, it also decreases Ex protein levels in the subapical cell cortex, indicating a post-transcriptional effect on Ex [27–30]. Consistent with the genetic epistatic evidence that places Ft upstream of Ex, we found that loss of *ft* induced *ex-lacZ* upregulation was significantly suppressed by co-depletion of *Bruce* (Fig. 2E-E’). Overexpression of *Dachs ligand with SH3s* (*Dlish*), an SH3 domain containing protein that physically interacts with Fat, could also cause overgrowth by destabilizing Ex [31]. Consistently, inhibition of *Bruce* significantly suppressed *Dlish*-induced wing overgrowth phenotype (Fig. S2D-D’).

### Bruce is required for ubiquitination-mediated Ex degradation

**Fig. 3:**
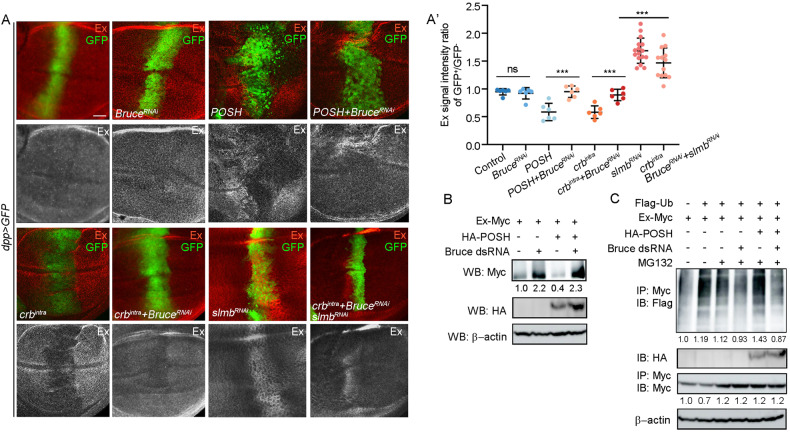
Bure regulates POSH-dependent Ex ubiquitination and degradation. **A** Fluorescent micrographs of wing pouch region with gene expression under the control of *dpp* promoter stained for Ex expression. Quantification of relative Ex signal intensity ratio of GFP^+^ region/GFP^−^ region (A’), *n* = 6, 6, 6, 6, 6, 6, 16, 13 (from left to right). **B** S2 cells transfected with HA-tagged POSH and Myc-tagged Ex were treated with or without dsRNAs of Bruce, and the lysates were analyzed by Western blotting. **C** Bruce is required for POSH-induced Ex ubiquitination in S2 cells. Mean ± SD; n.s. not significant; ****p < 0.0001; ordinary one-way ANOVA (A’). Scale bars: 20 μm for (**A**).

Our epistasis experiments placed Bruce downstream of Crb^intra^ and upstream of Ex to regulate Yki activation. Given that *crb* is known to function upstream in Hippo signaling, at least in part by regulating stability and localization of Ex [12–14, 16, 18, 20], we hypothesized that Bruce could modulate Ex stability. Firstly, we analyzed in vivo Ex protein level in the wing disc using immunostaining and did not observe distinct change upon *Bruce* depletion under the control of *dpp-Gal4* (Fig. 3A-A’). However, we noticed that although inhibition of endogenous *Bruce* is not sufficient to increase Ex abundance and accumulation at the apical junctional region in vivo (Fig. S3), strong upregulation of exogenously expressed Ex (Ex-Myc) was detected in *Drosophila* S2 cells treated with *Bruce* dsRNA (Fig. 3B). In accordance with this, knockdown of *Bruce* significantly suppressed *dpp* *>* crb^intra^-induced Ex degradation (Fig. 3A-A’). Furthermore, it has been reported that slmb, an E3 ubiquitin ligase, interacts with Crb to regulate the degradation of Ex [20]. Given that reducing *Bruce* alone did not fully reverse the decrease in Ex protein induced by crb^intra^, we simultaneously downregulated the expression of both *Bruce* and *slmb* in *dpp* *>* crb^intra^. Remarkably, this combined downregulation led to a significant increase in Ex protein level (Fig. 3A-A’).

As we have previously uncovered POSH as an Ex-binding E3 ligase that regulates Crb^intra^-induced Ex ubiquitination and degradation [15], we further explored the potential genetic interactions between POSH and Bruce. We found that POSH overexpression-induced Ex protein reduction phenotype was dramatically reversed by knocking down *Bruce* (Fig. 3A-A’). Furthermore, transfection of S2 cells with POSH-induced Ex ubiquitination and degradation were also suppressed by dsRNA-mediated *Bruce* knockdown (Fig. 3B, C), suggesting that ectopic POSH activity crucially depends on the E2 function of endogenous Bruce to regulate ubiquitination-mediated Ex degradation.

### Bruce synergizes with POSH to induce overgrowth and tumorigenesis

**Fig. 4:**
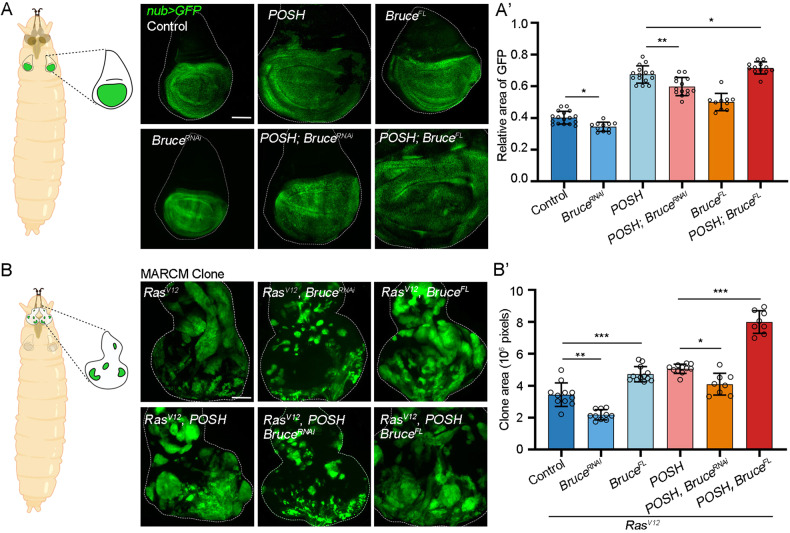
Bure synergizes with POSH to induce overgrowth and tumorigenesis. **A** Cartoon elucidation of wing discs from a *Drosophila* larva (left). GFP labeled wing pouch regions with indicated genotypes are shown (right). Quantification of relative GFP region size (A’), *n* = 17, 11, 15, 14, 10, 12 (from left to right). **B** Cartoon elucidation of eye-antennal discs from a *Drosophila* larva (left). Eye-antennal discs of *ey-Flp*-MARCM-induced GFP positive mosaic clones with indicated genotypes. Quantification of relative clone size (B’), *n* = 11, 10, 12, 11, 8, 8 (from left to right). Mean ± SD; *p < 0.05, **p < 0.01, ****p < 0.0001; ordinary one-way ANOVA (A’ and **B**). Scale bars: 100 μm for (**A**) and (**B**).

Next, we further explored the in vivo roles of *Bruce* and *POSH* in different contexts. Ectopic expression of POSH induces overgrowth phenotype in the wing epithelium under the control of *nub-Gal4*, which was significantly suppressed by depletion of *Bruce* (Fig. 4A-A’). Conversely, we noticed that co-expression of Bruce^FL^ and POSH synergistically induces wing overgrowth (Fig. 4A-A’). POSH is known to cooperate with oncogenic *Ras* (*Ras*^*V12*^) to induce tumor overgrowth and Hippo inactivation in the eye epithelium utilizing MARCM technique (Fig. 4B-B’) [32]. Similarly, we found that co-expression of Bruce^FL^ also synergistically enhances *Ras*^*V12*^-induced clonal overgrowth (Fig. 4B-B’). Consistent with the notion that *Bruce* is required for POSH induced growth in the wing disc, depletion of *Bruce* significantly inhibited *Ras*^*V12*^*/POSH*-induced tumor overgrowth in the eye epithelium (Fig. 4B-B’). Moreover, bioinformatic analysis of The Cancer Genome Atlas (TCGA) data revealed a high positive correlation across multiple human cancers between the mRNA level of *BIRC6* and *SH3RF1*, the human orthologs of *Bruce* and *POSH*, respectively (Fig. S4). Together, these data imply that Bruce collaborates with POSH to drive overgrowth and tumorigenesis.

### Bruce is essential for Hippo signaling-mediated malignant tumor progression

**Fig. 5:**
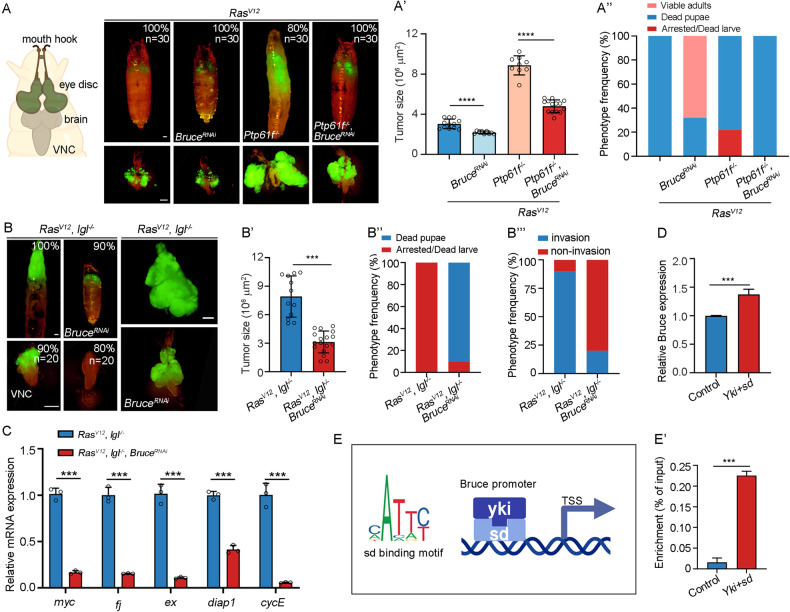
*Bruce* is required for Hippo pathway mediated malignant tumor progression. **A** Cartoon elucidation of a third-instar larvae cephalic complex (left). Dorsal views of *ey-Flp*-MARCM-induced GFP positive tumor bearing pupae or larvae (upper) and the corresponding cephalic complexes (bottom), % indicate representative phenotype penetrance. Quantification of tumor size (A’, *n* = 12, 9, 9, 14) and larvae phenotype (A”, *n* = 30, 30, 30, 30). **B** Dorsal views of *ey-Flp*-MARCM-induced GFP positive tumor bearing pupae or larvae (upper) and the corresponding ventral nerve cord (NVC) (bottom), % indicate representative phenotype penetrance. Quantification of tumor size (B’, *n* = 12, 17), larvae phenotype (B”, *n* = 20, 20), and invasion percentage (B”’, *n* = 20, 20). **C** qRT-PCR analysis of Yki target gene expression of tumors dissected from *Ras*^*V12*^*/lgl*^*−/−*^ and *Ras*^*V12*^*/lgl*^*−/−*^ *+* *Bruce*^*RNAi*^. **D** Quantification of relative *Bruce* mRNA level of S2 cells transfected with or without HA-Sd + Yki-Myc, *n* = 3. **E** Scheme of the *Bruce* promoter region and transcription start site (TSS), with the potential Sd binding motif (CATTT/C). (E’) S2 cells transfected with HA-Sd and Yki-Myc were used for Yki and Sd enrichment quantification on target region (−1326 to −1249) that contains Sd binding site, *n* = 3. Mean ± SD; *p < 0.05, ***p < 0.001, ****p < 0.0001; ordinary one-way ANOVA (A’, B’ and F’), two-tailed Student’s *t* tests (C’, D’ and E’). Scale bars: 500 μm for (**A**, **C**) (upper); 200 μm for (**A**, **C**) (bottom).

To further dissect the physiological function of Bruce during tumorigenesis, we depleted *Bruce* in several *Drosophila* malignant tumor models. Recently, we uncovered the tyrosine phosphatase *Ptp61F* as a crucial regulator of three-dimensional organ size under both physiological and pathological conditions and demonstrated that loss of *Ptp61F* synergizes with *Ras*^*V12*^ to induce Hippo-dependent tumorigenesis through Ex [33]. Clonal expression of *Ras*^*V12*^ alone in the eye-antennal disc induces hyperproliferation and forms benign tumors [32, 33], whereas simultaneously removal of *Ptp61F* results in malignant tumor formation (Fig. 5A-A’) [33], and 80% of the animals were unable to pupate or died as giant larvae (Fig. 5A”). Remarkably, we found that depletion of *Bruce* drastically inhibited the tumor overgrowth and related phenotypes (Fig. 5A-A”). Apart from *Ptp61F*, disruption of cell polarity gene *lethal (2) giant larvae* (*l(2)gl*) could also collaborate with *Ras*^*V12*^ to induce aggressive malignant tumors [34–36], with invasive migration into the ventral nerve cord (VNC) of the central nervous system (Fig. 5B). Consistently, inhibiting *Bruce* activity strongly impeded *Ras*^*V12*^*/lgl*^*−/−*^ triggered tumor overgrowth (Fig. 5B-B”), invasion (Fig. 5B”’), and transcriptional upregulation of multiple Yki target genes (Fig. 5C). Together, these data suggest that *Bruce* plays a significant role in the malignant tumor progression.

As multiple upstream components of Hippo pathway (e.g., Mer, Ex, Kibra, Ptp61F) can be transcriptionally activated by Yki/Sd to form a negative feedback loop to fine tune the pathway [4, 33], we examined whether *Bruce* could be a downstream target of Yki. Indeed, transfection of S2 cells with Yki and Sd significantly upregulated Bruce transcription level (Fig. 5D). Furthermore, our cleavage under targets and tagmentation (CUT&Tag) analysis in the S2 cells revealed that precipitated DNA was appreciably enriched in the promoter region of *Bruce* that contains putative Sd binding motif (Fig. 5E-E’). Collectively, these results demonstrate that Bruce is a direct transcriptional target of Yki/Sd.

### A putative conserved role of *BIRC6* in Hippo-dependent human cancer

**Fig. 6:**
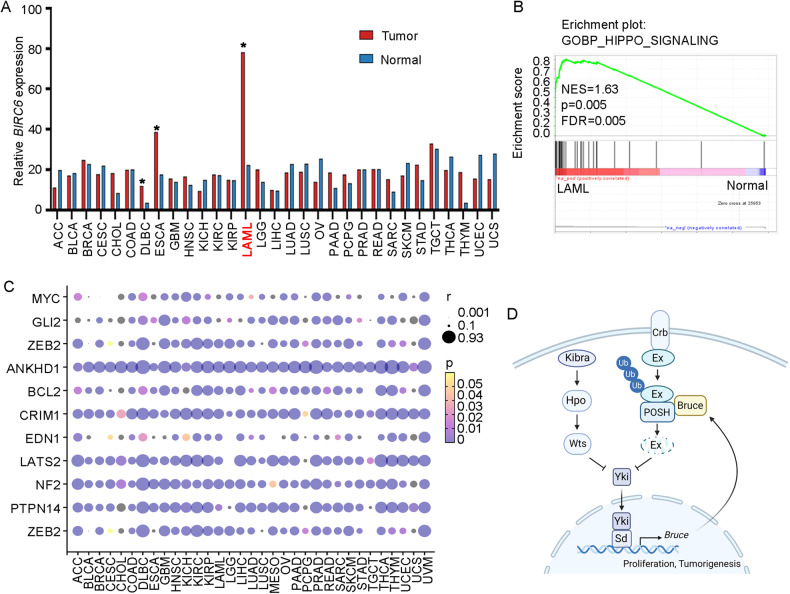
A putative conserved role of BIRC6 in Hippo-dependent human cancer. **A** Comparison of Bruce mRNA expression in different tumor samples (red) and paired normal tissues (blue) using the GEPIA2 (http://gepia2.cancer-pku.cn). **B** GSEA enrichment of Hippo pathway-related genes in LAML patients. **C** Heat map of the correlation coefficients between *Bruce* and multiple Hippo pathway target genes in TCGA patients, which are displayed in different colors and size. The size scale indicates the degree of correlation. The color of the points is based on the *p* value. **D** A model for Bruce-mediated Hippo signaling regulation.

As majority of Hippo pathway components are highly functional conserved between *Drosophila* and humans, we further explored whether Bruce-mediated Hippo signaling regulation is conserved during human tumor progression. We used GEPIA2 (Gene Expression Profiling Interactive Analysis, version 2, http://gepia2.cancer-pku.cn) [37] to analyze mRNA expression level of *BIRC6* (human *Bruce* homolog) in various human cancers, and found that *BIRC6* mRNA levels are strongly increased in several cancer type, with highest expression in acute myeloid leukemia (LAML) patients (Fig. 6A). Consistent with genetic correlation between *Bruce* and Hippo pathway in *Drosophila*, our gene set enrichment analysis (GSEA) results revealed a significant enrichment of genes associated with Hippo pathway in LAML patients (Fig. 6B). Moreover, we also observed strong positive correlations between the mRNA of *BIRC6* and multiple Hippo pathway target genes across human cancers (Fig. 6C). Collectively, these data indicate that BIRC6 might play a conserved role in regulating Hippo pathway in human cancers.

## Discussion

Ubiquitination is an essential post-translational modification process that determines protein stability or subcellular localization, dysregulation of which affects various cellular activities, including cell cycle, cell death, and tumorigenesis [38]. Despite the identification of numerous E3 ligases as regulators of key Hippo pathway components [7–11, 15, 20, 39], the responsible E2 that regulates ubiquitin-mediated Hippo signaling activation remains unknown. Here, we have conducted an RNAi screen against E2 coding genes in *Drosophila* and found that Bruce positively regulates the Hippo pathway by genetically acting upstream of Ex and in parallel with Hpo-Wts cascade (Fig. 6D). First, we demonstrate that depletion of *Bruce* downregulated endogenous Yki target gene expression and suppressed Crb^intra^-induced tissue growth. Second, our genetic epistasis data indicate that Bruce acts upstream of Ex but in parallel with the conical Kibra-Hpo-Wts cascade. Third, we show that Bruce is required for POSH overexpression induced Ex ubiquitination and degradation and reveal a collaborative role between POSH and Bruce in growth control. We noticed that depletion of Bruce alone did not significant impact the level of endogenous Ex protein, suggesting that Bruce may play a pivotal role in stress or pathological conditions. It is probable that Bruce actively engages in cellular stress response mechanisms to maintain or restore the normal levels of Ex protein. Consequently, additional studies should be carried out to explore the interaction mechanism between Bruce and Ex, as well as their specific functions in regulating Ex protein degradation. Apart from being a master regulator of organ growth, the Hippo signaling pathway has been implicated in various cancer development in mice and humans. As in mammals, Hippo pathway-dependent tumors can be genetically induced in various *Drosophila* tissues, including eye epithelium, gut, and ovary [40, 41]. For instance, overexpression of *Ras*^*V12*^ in *lgl* mutant clones (*Ras*^*V12*^*/lgl*^*−/−*^) in eye-antennal discs resulted in tumor-like growth with invasive migration into adjacent regions [32, 35, 36]. We show that inhibition of *Bruce* dramatically impeded *Ras*^*V12*^*/lgl*^*−/−*^ triggered tumor overgrowth, invasion, as well as activation of Hippo pathway target genes. Similarly, *Ras*^*V12*^*/Ptp61F*^*−/−*^-induced malignant tumor progression was also suppressed by knocking down *Bruce*, suggesting that *Bruce* is a putative oncogene. Remarkably, a recent study demonstrated that the Bruce’s homolog BIRC6 is selectively required for the survival of a subset of epithelial tumors with a high degree of aneuploidy, and *BIRC6* inhibition reduced cell fitness by activating the integrated stress response (ISR) [42]. Consistent with *BIRC6*’s role as a putative oncogene [43, 44], we observe strong enrichment of Hippo signature genes in LAML patients and reveal intensively strong correlations between *BRIC6* mRNA and Hippo pathway target genes across numerous cancer types. Collectively, given the conservation of Hippo pathway and overwhelming evidence that *Drosophila* is an excellent model to study human cancer biology [4, 40, 41, 45], our findings here indicate that similar mechanisms might exist between the E2 enzyme BIRC6 and Hippo signaling in mammals during both normal development and cancer progression.

## Materials and methods

### Key resource table

**Table:** 

Reagent or resource	Source	Identifier
Antibodies		
mouse monoclonal anti-β-gal	Developmental Studies Hybridoma Bank	Cat# 40-1a; RRID: AB_2314509
rat monoclonal anti-Ci	Developmental Studies Hybridoma Bank	Cat# 2A1; RRID: AB_2109711
rabbit polyclonal anti-Phospho-Histone H3	Cell Signaling Technology	Cat# 9701; RRID: AB_331535
rabbit polyclonal anti- Cleaved *Drosophila* Dcp-1	Cell Signaling Technology	Cat# 9578; RRID: AB_2721060
HA-Tag (C29F4) Rabbit	Cell Signaling Technology	Cat# 3724; RRID: AB_1549585
Myc-tag mouse monoclonal	Cell Signaling Technology	Cat# 2276; RRID: AB_331783
mouse monoclonal anti-β-actin	Cell Signaling Technology	Cat# 4968, RRID: AB_2313904
guinea-pig monoclonal anti-Ex	Gift from Richard Fehon (University of Chicago)	Maitra et al. [46]
Ubiquitin Antibody Rabbit	Cell Signaling Technology	Cat# 3933, RRID: AB_2180538
Goat anti-Mouse IgG (H + L) Alexa Fluor^TM^ Plus 555	Invitrogen	Cat# A32727; RRID: AB_2633276
Goat anti-Rabbit IgG (H + L) Alexa Fluor^TM^ Plus 555	Invitrogen	Cat# A32732; RRID: AB_2633281
Chemicals, peptides, and recombinant proteins		
Trizol	Thermo Fisher Scientific	Cat# 15596026
Antifade Mounting Medium with DAPI	Vector Laboratories	Cat# H-1800
Critical commercial assays		
ESF 921 Insect Cell Culture Medium	Expression System	Cat# 96-001-01
Fetal bovine Serum	CellMax	Cat# SA112
Penicillin-Streptomycin (100X)	Thermo Fisher Scientific	Cat# 15070063
Antibiotic-Antimycotic	Thermo Fisher Scientific	Cat# 15240096
Effectene Transfection Reagent	QIAGEN	Cat# 301427
Protease inhibitor cocktail	Sigma	Cat# P8340
BCA Protein Quantification Kit	Vazyme	Cat# E112-01
PVDF membrane	Merck Millipore	Cat# IPVH00010
Hyperactive Universal CUT&Tag Assay Kit	Vazyme	Cat# TD903-00
Taq Pro Universal SYBR qPCR Master Mix	Vazyme	Cat# Q712-02
MEGAscript^TM^ RNAi Kit	Invitrogen	Cat# AM1626
Pierce™ Protein A/G Magnetic Beads	Thermo Fisher Scientific	Cat# 88802
FastPure Plasmid Mini Kit	Vazyme	Cat# DC201-01
HiScript II 1st Strand cDNA Synthesis Kit	Vazyme	Cat# R211-01
Oligonucleotides		
qPCR for *Bruce* F	GGGTGACTTTCTCGCTGACA	N/A
qPCR for *Bruce* R	CCCAGCGAGCTGCTGATTTA	N/A
qPCR for CUT&Tag *Bruce* F	TTTAAGCGGAATTATCGCG	N/A
qPCR for CUT&Tag *Bruce* R	CCAGTTATTCAACCGTTAC	N/A
DsRNA for *Bruce* F	TAATACGACTCACTATAGGGAGAGCATGAAGACCTGTGTGGAT	N/A
Oligonucleotides		
DsRNA for *Bruce* R	CTCCCTATAGTGAGTCGTATTATACAGAACTACTAAAGCTGAGG	N/A
qPCR for *rp49* F	CCACCAGTCGGATCGATATGC	N/A
qPCR for *rp49* R	CTCTTGAGAACGCAGGCGACC	N/A
qPCR for *ex* F	GGATCTACCGTAAGCCACCG	N/A
qPCR for *ex* R	TCATCATGACAGCGGGATCG	N/A
qPCR for *diap1* F	AAATGCTTTTTCTGCGGCGT	N/A
qPCR for *diap1* R	CTCATCTCCAGCGTCGAGTC	N/A
qPCR for *fj* F	AGGGATGCGGAAGAATGCAA	N/A
qPCR for *fj* R	GGCTGAAAGGGCTGTGGTAT	N/A
qPCR for *myc* F	AGCCAGAGATCCGCAACATC	N/A
qPCR for *myc* R	CGCGCTGTAGAGATTCGTAGAG	N/A
qPCR for *cycE* F	GAATGGAGAGCGTACTCCCG	N/A
qPCR for *cycE* R	TCCAAGAGAATGGCACGCAT	N/A
Experimental models: Cell lines		
*Drosophila* S2 cells	Gift from Jose’ C. Pastor-Pareja (Tsinghua University)	FBtc9000001
Experimental models: Organisms/strains		
The *Drosophila* strains used in this study are listed in Supplementary Table 1	N/A	N/A
Software and algorithms		
GraphPad Prism 8.0	GraphPad Software	RRID: SCR_002798
Fiji/ImageJ software	https://fiji.sc	Schindelin et al. [47]
Adobe Photoshop	https://www.adobe.com/cn/products/photoshop.	RRID: SCR_014199

### Fly stocks and genetics

*Drosophila* stocks and crosses were raised on standard cornmeal-yeast-agar medium at 25 °C. The standard cornmeal-yeast food including 9 g agar, 24.5 g dry yeast, 50 g corn flour, 7.25 g white sugar, 4.4 ml propionic acid, 30 g brown sugar, 1.25 g nipagin per liter and 12.5 ml ethanol. All *Drosophila* lines used in this study were listed and described in Supplementary Table 1, and detailed genotypes for each figure are described in Supplementary data.

Fluorescently labeled mitotic clones were produced in the eye-antennal discs using the mosaic analysis with a repressible marker (MARCM) system with the following strains: y, w, eyFLP1; Act5C > y + > Gal4, UAS-GFP; FRT82B, Tub-Gal80 (82B tester); yw, ey-Flp; act > y + > GAL4, UAS-GFP; Tub-GAL80, FRT 80B (80B tester); yw, ey-Flp; tub-Gal80, FRT 40 A; act > y + > GAL4 UAS-GFP (40A tester).

### *Drosophila* cell culture

*Drosophila* S2 cells were cultured at 25 °C in an atmosphere and in Cell Culture Medium supplemented with 10% fetal bovine serum and 1% Antibiotic-Antimycotic.

### Cell transfection

Plasmid transfection and double-stranded RNA (dsRNA) were conducted using Effectene transfection reagent according to the manufacturer’s instructions. Plasmids used for Immunoprecipitation (IP) were: pUAST-attB-HA-POSH; pUAST-attB-Ex-Flag; pUAST-Ubi-myc. Plasmids used for Cut&Tag assay were: pUAST-HA-Sd and pUAST-Yki-myc. dsRNA of *Bruce* was prepared with MEGAscript^TM^ RNAi Kit according to the manufacturer’s instructions, and primers listed in Oligonucleotides.

### IP assay and western blot

Cells were crushed with NP40 buffer (50 mM Tris (pH7.4), 150 mM NaCl, 1% NP-40, sodium pyrophosphate, β-glycerophosphate, sodium orthovanadate, sodium fluoride, EDTA and leupeptin). Cell lysate and pre-washed magnetic beads incubate with rotation for 20 min at room temperature. Separate the beads from the lysate using a magnetic separation rack, transfer the pre-cleared lysate to a clean tube. Then, add primary antibody to 200 μl cell lysate, and incubate overnight at 4 °C. After that, pre-washed magnetic bead was added to the lysate and antibody solution, Finally, the bound protein can be finally separated through the magnetic beads. Proteins were quantitated by using the BCA according to the manufacture’s instruction, followed by separation with SDS-PAGE and transferred to PVDF membranes. The membranes were blocked for 60 min with 5% bovine serum albumin in TBST and blotted with the following primary antibodies for 12–16 h at 4 °C: β-actin, HA, Flag and Myc. After washing with TBST three times, membranes were incubated with HRP-conjugated secondary antibodies for visualization.

### Cut & Tag assay

Cut & Tag assay was performed with Hyperactive Universal CUT&Tag Assay Kit according to the manufacturer’s instructions. Briefly, S2 cells are bound by magnetic beads (Concanavalin A-coated Magnetic Beads Pro, ConA Beads Pro) and membrane permeabilization is performed using the nonionic detergent digitonin. Through the mediation of the primary antibody (Rabbit IgG or HA-tag or Mouse myc-tag), corresponding secondary antibody and Protein A/G against the target protein, transposons fused with Protein A/G can precisely target DNA sequences near the target protein. And enrichment of DNA sequences was detected using qPCR and primers listed in Oligonucleotides. Data were normalized and analyzed using fold enrichment analysis.

### RNA isolation and qRT-PCR

Total RNA that extracted from S2 cell line was using Trizol. For mRNA expression analysis, the first-strand cDNA was generated using HiScript II 1st Strand cDNA Synthesis Kit. Quantitative Real-time PCR (q-PCR) was performed on triplicate samples in a reaction mix of Taq Pro Universal SYBR qPCR Master Mix with Jena Qtower384G Real-Time PCR System

### Immunofluorescence, immunohistochemistry, and imaging

Third-instar larvae imaginal disc were dissected in cold PBS and fixed with 4% paraformaldehyde for 15 min at room temperature, then washed with PBS containing 0.1% Triton X-100 solution (PBST) for 5 min, three times. Samples were blocked in PBS with 5% normal donkey serum for 1 h and then incubated with primary antibodies at 4 °C overnight. After that, samples were incubated with secondary antibodies for 2 h at room temperature, then mounted with DAPI-containing medium. The images were performed with Zeiss Axio Observer with ApoTome.2. Images were processed with Fiji/Image J software (https://fiji.sc), Photoshop CS8 (Abode) for image merging and resizing.

### Gene set enrichment analysis (GSEA)

GSEA analysis was performed with GSEA software (http://www.broadinstitute.org/gsea/index.jsp), using differentiation gene sets in LAML (Acute Myeloid Leukemia) and normal samples from TCGA (https://portal.gdc.cancer.gov/). GSEA results were considered significant when the FDR *q* value was less than 0.25 and *p* value was less than 0.05.

### Quantification and statistical analysis

For clone-size quantification of wild type or mutant, the acquired pictures using fluorescence microscope were analyzed using ImageJ. Clone size was measured as “total GFP clone area/disc area (%)” by analyzing all the clones in at least 8 randomly selected eye discs (~10 discs on average) of each genotype. For antibody staining quantification within GFP or wild-type cells, a minimum of 10 wing discs was analyzed in ImageJ for the mean signal intensity of GFP cells versus neighboring wild-type cells. For quantification of adult eye phenotype, the eye area of female flies was measured manually using ImageJ.

Survival analysis was performed to investigate the associations between the censored outcomes and the expression measures of BIRC6 using GEPAI (http://gepia.cancer-pku.cn/). Pearson correlation analysis was employed to determine the correlation between the expression of Hippo targets and BIRC6 from TCGA. Statistical analyses were performed with GraphPad Prism 9.0. software. Unless indicated, statistical significance was calculated by unpaired, two-tailed Student’s *t* tests, ordinary ANOVA test (Tukey’s multiple comparisons test) for three or more groups and Mann–Whitney *U* tests. The number of animals used per experiment can be found in the figure legends. Data are mean ± s.d. ns (not significant), *p ≤ 0.05, **p ≤ 0.01, ***p < 0.001, ****p < 0.0001. *n* ≥ 3 independent experiments.

## Supplementary information

Supplemental Table

Supplemental data

original data files

aj-checklist

## Data Availability

The authors confirm that the data supporting the findings of this study are available within the article.
