# Supplementary material for: E2 enzyme Bruce negatively regulates Hippo signaling through POSH-mediated expanded degradation
Source: Cell Death Dis. 2023 Sep 12;14(9):602. doi: 10.1038/s41419-023-06130-2 (PMC10497580; doi:10.1038/s41419-023-06130-2)
Supplement: Supplementary file 2 — Supplemental data [file 41419_2023_6130_MOESM2_ESM.docx]

**E2 enzyme Bruce negatively Regulates Hippo Signaling Through POSH-Mediated Expanded Degradation**

Sha Song & Xianjue Ma

Supplementary information contains the following sections:

Detailed genotypes for all the figures;

Supplemental Figs 1-4 and related figure legends;

**Detailed genotypes**.

The detailed genotypes of each experiment are described below:

| **Figure 1** |  |
| --- | --- |
| **Fig. 1A** | *w; nub-Gal4/+* |
|  | *w; nub-Gal4, UAS-crb^intra^/+* |
|  | *w; nub-Gal4, UAS-crb^intra^/UAS-vih^RNAi^* |
|  | *w; nub-Gal4, UAS-crb^intra^/UAS-CG16894^RNAi^* |
|  | *w; nub-Gal4, UAS-crb^intra^/+; UAS-CG5823^RNAi^/+* |
|  | *w; nub-Gal4, UAS-crb^intra^/+; UAS-Bruce^RNAi^/+* |
|  | *w; nub-Gal4, UAS-crb^intra^/UAS-CG2924^RNAi^* |
|  | *w; nub-Gal4, UAS-crb^intra^/+; UAS-Ubc84D^RNAi^/+* |
|  | *w; nub-Gal4, UAS-crb^intra^/UAS-CG8188^RNAi^* |
|  | *w; nub-Gal4, UAS-crb^intra^/+; UAS-Ubc10^RNAi^/+* |
|  | *w; nub-Gal4, UAS-crb^intra^/UAS-Ubc4^RNAi^* |
|  | *w; nub-Gal4, UAS-crb^intra^/+; UAS-CG7656^RNAi^/+* |
|  | *w; nub-Gal4, UAS-crb^intra^/+; UAS-CG5440^RNAi^/+* |
|  | *w; nub-Gal4, UAS-crb^intra^/+; UAS-ben^RNAi^/+* |
|  | *w; nub-Gal4, UAS-crb^intra^/UAS-Ubc7^RNAi^* |
|  | *w; nub-Gal4, UAS-crb^intra^/UAS-CG10254^RNAi^* |
|  | *w; nub-Gal4, UAS-crb^intra^/UAS-CG14739^RNAi^* |
|  | *w; nub-Gal4, UAS-crb^intra^/+; UAS-Ubc6^RNAi^/+* |
|  | *w; nub-Gal4, UAS-crb^intra^/UAS-CG17030^RNAi^* |
|  | *w; nub-Gal4, UAS-crb^intra^/UAS-CG40045^RNAi^* |
|  | *w; nub-Gal4, UAS-crb^intra^/UAS-CG10862^RNAi^* |
|  | *w; nub-Gal4, UAS-crb^intra^/+; UAS-CG7220^RNAi^/+* |
|  | *w; nub-Gal4, UAS-crb^intra^/UAS-UbcE2H^RNAi^* |
|  | *w; nub-Gal4, UAS-crb^intra^/+; UAS-UbcE2M^RNAi^/+* |
|  | *w; nub-Gal4, UAS-crb^intra^/+; UAS-CG3473^RNAi^/+* |
|  | *w; nub-Gal4, UAS-crb^intra^/UAS-Ubc2^RNAi^* |
|  | *w; nub-Gal4, UAS-crb^intra^/+; UAS-CG2574^RNAi^/+* |
|  | *w; nub-Gal4, UAS-crb^intra^/+; UAS-CG4502^RNAi^/+* |
|  | *w; nub-Gal4, UAS-crb^intra^/+; UAS-eff^RNAi^/+* |
|  | *w; nub-Gal4, UAS-crb^intra^/+; UAS-lwr^RNAi^/+* |
|  | *w; nub-Gal4, UAS-crb^intra^/+; UAS-Ubc87F^RNAi^/+* |
| **Fig. 1B and C** | *w; nub-Gal4, UAS-GFP/+* |
|  | *w; nub-Gal4, UAS-GFP, UAS-crb^intra^/+* |
|  | *w; nub-Gal4, UAS-GFP/+; UAS-Bruce^RNAi^/+* |
|  | *w; nub-Gal4, UAS-GFP, UAS-crb^intra^/+; UAS-Bruce^RNAi^/+* |
| **Fig. 1D** | *w; GMR-Gal4/+* |
|  | *w; GMR-Gal4/UAS-crb^intra^* |
|  | *w; GMR-Gal4/+; UAS-Bruce^RNAi^/+* |
|  | *w; GMR-Gal4/UAS-crb^intra^; UAS-Bruce^RNAi^/+* |
| **Fig. 1E** | *w;* *ex^[e1]^/+; dpp>GFP/+* |
|  | *w;* *ex^[e1]^/UAS-crb^intra^; dpp>GFP/+* |
|  | *w;* *ex^[e1]^/UAS-crb^intra^; dpp>GFP/UAS-Bruce^RNAi^* |
| **Fig 1F** | *w; ex^[e1]^/+; hh-Gal4, UAS-GFP/+* |
|  | *w;* *ex^[e1]^/+; hh-Gal4, UAS-GFP/UAS-Bruce^RNAi^* |
|  | *w;* *en-Gal4, UAS-GFP/ fj^[9-II]^* |
|  | *w;* *en-Gal4, UAS-GFP/fj^[9-II]^; UAS-Bruce^RNAi^/+* |
|  | *w;* *en-Gal4, UAS-GFP /+; diap1^j5C8^/+* |
|  | *w;* *en-Gal4, UAS-GFP /+; diap1^j5C8^/UAS-Bruce^RNAi^* |
|  | *w;; hh-Gal4, UAS-GFP /+* |
|  | *w;; hh-Gal4, UAS-GFP/UAS-Bruce^RNAi^* |
| **Figure 2** |  |
| **Fig. 2A** | *w; nub-Gal4/+* |
|  | *w; nub-Gal4/+; UAS-Bruce^RNAi^/+* |
|  | *w; nub-Gal4/UAS-ex^RNAi^* |
|  | *w; nub-Gal4/UAS-ex^RNAi^; UAS-Bruce^RNAi^/+* |
|  | *w; nub-Gal4/UAS-kibra^RNAi^* |
|  | *w; nub-Gal4/UAS-kibra^RNAi^; UAS-Bruce^RNAi^/+* |
|  | *w; nub-Gal4/UAS-hpo^RNAi^* |
|  | *w; nub-Gal4/UAS-hpo^RNAi^; UAS-Bruce^RNAi^/+* |
|  | *w; nub-Gal4/UAS-ft^RNAi^* |
|  | *w; nub-Gal4/UAS-ft^RNAi^; UAS-Bruce^RNAi^/+* |
| **Fig. 2B** | *w; nub-Gal4, UAS-GFP/UAS-crb^intra^* |
|  | *w; nub-Gal4, UAS-GFP/UAS-wts^RNAi^; UAS-Bruce^RNAi^/+* |
|  | *w; nub-Gal4, UAS-GFP/UAS-yki* |
|  | *w; nub-Gal4, UAS-GFP/UAS-yki; UAS-Bruce^RNAi^/+* |
| **Fig. 2C** | *w; ex^[e1]^/UAS-ft^RNAi^; hh>GFP/+* |
|  | *w;* *ex^[e1]^/UAS-ft^RNAi^; hh>GFP/UAS-Bruce^RNAi^* |
|  | *w; ex^[e1]^/UAS-scrib^RNAi^; hh-Gal4/+* |
|  | *w;* *ex^[e1]^/UAS-scrib^RNAi^; hh-Gal4/UAS-Bruce^RNAi^* |
| **Fig. 2D** | *ey-Flp1/+; FRT40A/Tub-Gal80, FRT40A;* *Act5C>y^+^>Gal4, UAS-GFP/+* |
|  | *ey-Flp1/+; FRT40A/Tub-Gal80, FRT40A;* *Act5C>y^+^>Gal4, UAS-GFP/Bruce^RNAi^* |
|  | *ey-Flp1/+; FRT40A, ex^[e1]^/Tub-Gal80, FRT40A;* *Act5C>y^+^ >Gal4, UAS-GFP/+* |
|  | *ey-Flp1/+; FRT40A, ex^[e1]^/Tub-Gal80, FRT40A;* *Act5C>y^+^>Gal4, UAS-GFP/UAS-Bruce^RNAi^* |
|  | *ey-Flp1/+; FRT42D, hpo^[42-47]^/Tub-Gal80, FRT42D;* *Act5C>y^+^>Gal4, UAS-GFP/+* |
|  | *ey-Flp1/+; FRT42D, hpo^[42-47]^/Tub-Gal80, FRT42D;* *Act5C>y^+^>Gal4, UAS-GFP/UAS-Bruce^RNAi^* |
|  | *ey-Flp1/+; Act5C>y^+^>Gal4, UAS-GFP/+ ; FRT82B, Tub-Gal80/FRT82B,wts^[X1]^* |
|  | *ey-Flp1/+; Act5C>y^+^>Gal4, UAS-GFP/UAS-Bruce^RNAi^ ; FRT82B, Tub-Gal80/FRT82B,wts^[X1]^* |
|  | *ey-Flp1/+; FRT42D, UAS-myc-yki/Tub-Gal80, FRT42D;* *Act5C>y^+^>Gal4, UAS-GFP/+* |
|  | *ey-Flp1/+; FRT42D, UAS-myc-yki/Tub-Gal80, FRT42D;* *Act5C>y^+^>Gal4, UAS-GFP/UAS-Bruce^RNAi^* |
| **Fig. 2E** | *w; ex^[e1]^/UAS-wts^RNAi^; dpp>GFP/+* |
|  | *w; ex^[e1]^/UAS-wts^RNAi^; dpp>GFP/UAS-Bruce^RNAi^* |
| **Figure 3** |  |
| **Fig. 3A** | *w;; dpp>GFP/+* |
|  | *w;; dpp>GFP/UAS-Bruce^RNAi^* |
|  | *w;UAS-POSH/+; dpp>GFP/+* |
|  | *w;UAS-POSH/+; dpp>GFP/UAS-Bruce^RNAi^* |
|  | *w;UAS-crb^intra^/+; dpp>GFP/+* |
|  | *w;UAS-crb^intra^/+; dpp>GFP/UAS-Bruce^RNAi^* |
|  | *w;UAS-slmb^RNAi^; dpp>GFP/+* |
|  | *w; UAS-crb^intra^/UAS-slmb^RNAi^; dpp>GFP/UAS-Bruce^RNAi^* |
| **Figure 4** |  |
| **Fig. 4A** | *w; nub-Gal4, UAS-GFP/+* |
|  | *w; nub-Gal4, UAS-GFP/UAS-POSH* |
|  | *w; nub-Gal4, UAS-GFP/UAS-Bruce^FL^* |
|  | *w; nub-Gal4, UAS-GFP/+;UAS-Bruce^RNAi^/+* |
|  | *w; nub-Gal4, UAS-GFP/UAS-POSH; UAS-Bruce^RNAi^/+* |
|  | *w; nub-Gal4, UAS-GFP/UAS-POSH, UAS-Bruce^FL^* |
| **Fig. 4B** | *ey-Flp1/+; Act5C>y^+^>Gal4, UAS-GFP/+; Tub-Gal80, FRT79E/ FRT79E, UAS-Ras^V12^* |
|  | *ey-Flp1/+; Act5C>y^+^>Gal4, UAS-GFP/UAS-Bruce^RNAi^; Tub-Gal80, FRT79E/ FRT79E, UAS-Ras^V12^* |
|  | *ey-Flp1/+; Act5C>y^+^>Gal4, UAS-GFP/UAS-Bruce^FL^; Tub-Gal80, FRT79E/ FRT79E, UAS-Ras^V12^* |
|  | *ey-Flp1/+; Act5C>y^+^>Gal4, UAS-GFP/UAS-POSH; Tub-Gal80, FRT79E/ FRT79E, UAS-Ras^V12^* |
|  | *ey-Flp1/+; Act5C>y^+^>Gal4, UAS-GFP/UAS-POSH, UAS-Bruce^RNAi^; Tub-Gal80, FRT79E/ FRT79E, UAS-Ras^V12^* |
|  | *ey-Flp1/+; Act5C>y^+^>Gal4, UAS-GFP/UAS-POSH, UAS-Bruce^FL^; Tub-Gal80, FRT79E/ FRT79E, UAS-Ras^V12^* |
| **Figure 5** |  |
| **Fig. 5A** | *ey-Flp1/+*; *Act5C>y^+^>Gal4*, UAS-GFP/*UAS-Ras^V12^*; *Tub-Gal80, FRT 80B/ FRT 80B* |
|  | *ey-Flp1/+*; *Act5C>y^+^>Gal4*, UAS-GFP/*UAS-Ras^V12^, UAS-Bruce^RNAi^*; *Tub-Gal80, FRT 80B/ FRT 80B* |
|  | *ey-Flp1/+*; *Act5C>y^+^>Gal4*, *UAS-GFP*/*UAS-Ras^V12^*; *Tub-Gal80, FRT 80B/Ptp61F^Δ^, FRT 80B*; |
|  | *ey-Flp1/+*; *Act5C>y^+^>Gal4*, *UAS-GFP*/*UAS-Ras^V12^, UAS-Bruce^RNAi^*; *Tub-Gal80, FRT 80B/Ptp61F^Δ^, FRT 80B* |
| **Fig. 5B** | *ey-Flp1/+*; *lgl^4^, FRT40A, UAS-Ras^V12^ /Tub-Gal80, FRT40A; Act5C>y^+^>Gal4, UAS-GFP/+* |
|  | *ey-Flp1/+*; *lgl^4^, FRT40A, UAS-Ras^V12^ /Tub-Gal80, FRT40A; Act5C>y^+^>Gal4, UAS-GFP/UAS-Bruce^RNAi^* |
| **Figure S1** |  |
| **Fig. S1A and B** | *w; nub-Gal4, UAS-GFP/+* |
|  | *w; nub-Gal4, UAS-GFP, UAS-crb^intra^/+* |
|  | *w; nub-Gal4, UAS-GFP/UAS-Bruce^RNAi^* |
|  | *w; nub-Gal4, UAS-GFP, UAS-crb^intra^/UAS-Bruce^RNAi^* |
| **Fig. S1C** | *w; GMR-Gal4/+* |
|  | *w; GMR-Gal4/UAS-crb^intra^* |
|  | *w; GMR-Gal4/UAS-Bruce^RNAi^* |
|  | *w; GMR-Gal4/UAS-crb^intra^, UAS-Bruce^RNAi^* |
| **Figure S2** |  |
| **Fig. S2A** | *w; GMR-Gal4^S^/+* |
|  | *w; GMR-Gal4^S^/UAS-Bruce^FL^* |
| **Fig. S2B** | *w; GMR-Gal4, UAS-Hpo/+* |
|  | *w; GMR-Gal4, UAS-Hpo/UAS-Bruce^FL^* |
| **Fig. S2C** | *ey-Flp1/+; FRT40A/Tub-Gal80, FRT40A;* *Act5C>y^+^>Gal4, UAS-GFP/+* |
|  | *ey-Flp1/+; FRT40A, UAS-Bruce^FL^/Tub-Gal80, FRT40A;* *Act5C>y^+^ >Gal4, UAS-GFP/+* |
|  | *ey-Flp1/+; Act5C>y^+^>Gal4, UAS-GFP/+; FRT82B, Tub-Gal80/FRT82B,* *kibra^Δ^* |
|  | *ey-Flp1/+; Act5C>y^+^>Gal4, UAS-GFP/UAS-Bruce^FL^; FRT82B, Tub-Gal80/FRT82B, kibra^Δ^* |
|  | *ey-Flp1/+; FRT40A, ex^[e1]^/Tub-Gal80, FRT40A;* *Act5C>y^+^ >Gal4, UAS-GFP/+* |
|  | *ey-Flp1/+; FRT40A, ex^[e1]^, UAS-Bruce^FL^ /Tub-Gal80, FRT40A;* *Act5C>y^+^ >Gal4, UAS-GFP/+* |
| **Fig. S2D** | *w; nub-Gal4/+* |
|  | *w; nub-Gal4/+; UAS-Bruce^RNAi^/+* |
|  | *w; nub-Gal4/UAS-Dlish* |
|  | *w; nub-Gal4/UAS-Dlish; UAS-Bruce^RNAi^/+* |
| **Figure S3** | *w;; hh-Gal4, UAS-GFP/UAS-Bruce^RNAi^* |


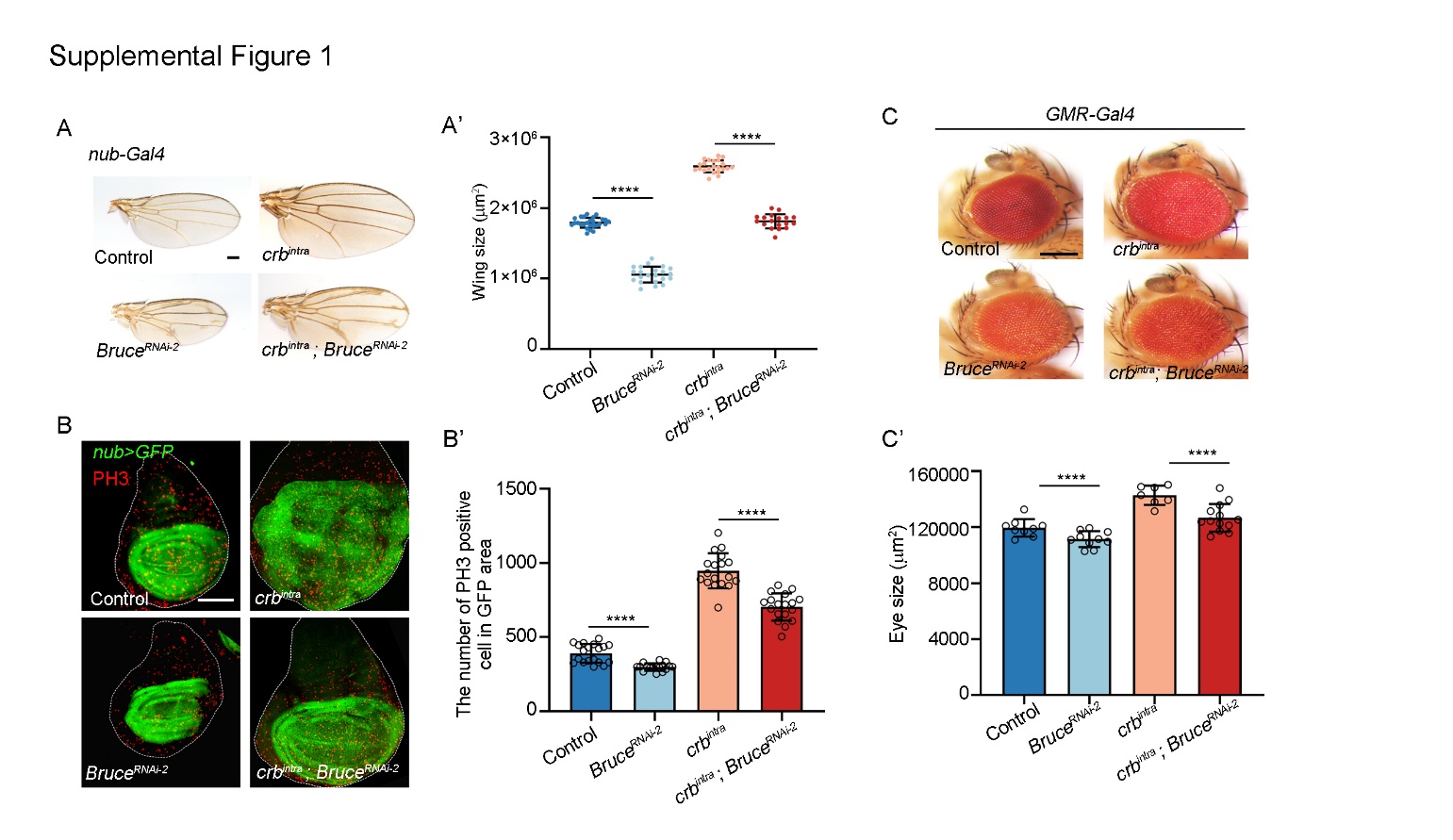


**Supplementary Figure 1. *Bruce* is required for ectopic expression of *crb^intra^*-induced growth.**

(**A**) Light micrographs of adult wings bearing indicated genotypes are shown. Quantification of wing size (A’), n = 26, 22, 22, 18 (from left to right). (**B**) GFP labeled wing discs bearing indicated genotypes. (B’) Quantification of PH3 positive cells of indicated genotypes, n = 21, 18, 29, 20 (from left to right). (**C**) Light micrographs of adult eyes bearing indicated genotypes are shown. Quantification of eye area (C’), n =9, 10, 7, 13 (from left to right). Mean ± SD; *****P* < 0.0001; ordinary one-way ANOVA tests (A’, B’ and C’). Scale bars, 200 μm for A and C; 100 μm for B.

**
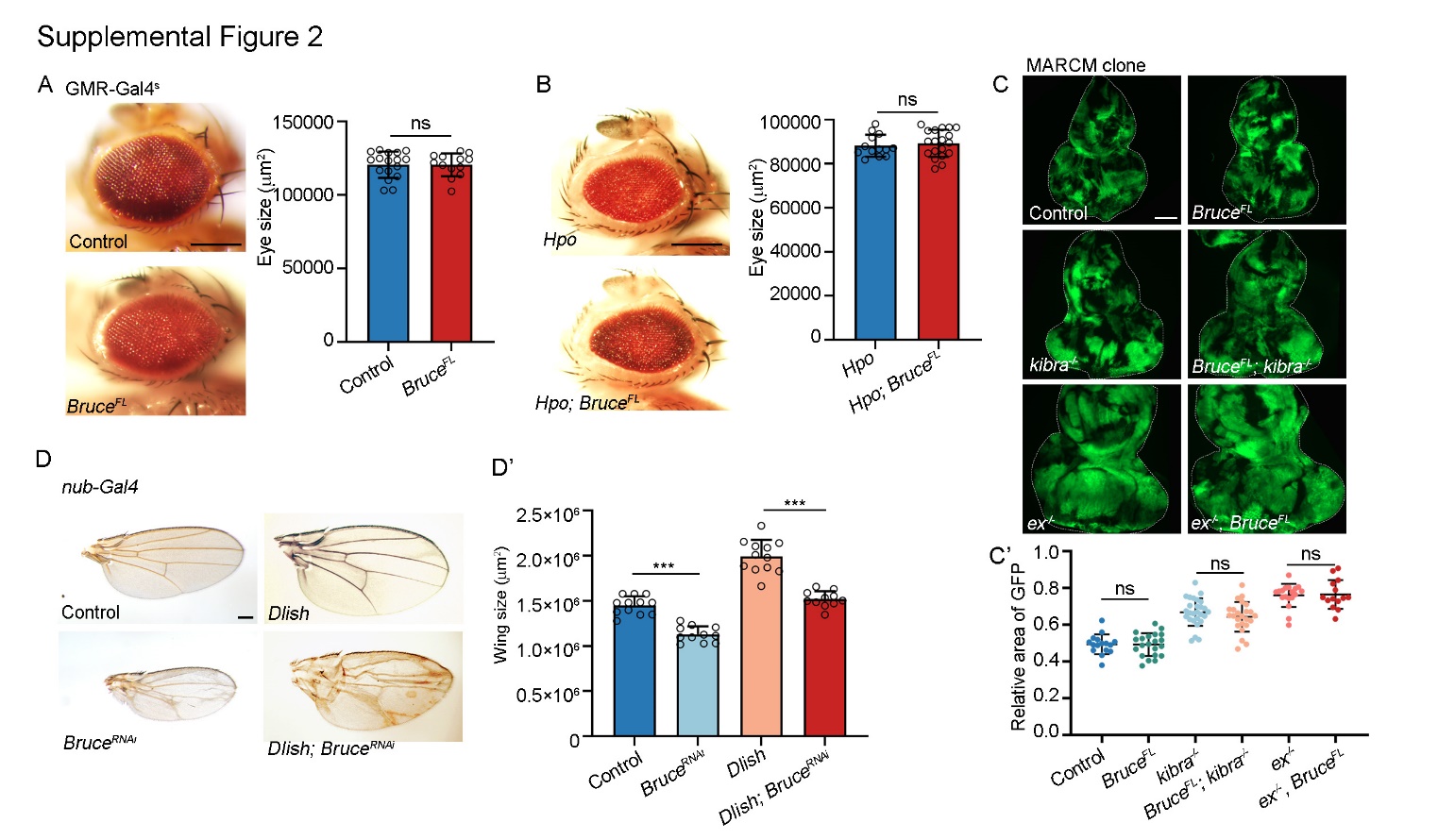
**

**Supplementary Figure 2. *Bruce* is required for *Dlish* expression induced overgrowth.**

(**A** and **B**) Light micrographs of adult eyes bearing indicated genotypes are shown. Quantification of eye area (A’, n=17, 13; B’, n =13, 19). (**C**) Eye-antennal discs of ey-Flp-MARCM-induced GFP positive mosaics clones with indicated genotypes. Quantification of clone size was shown in C’, n = 16, 21, 27, 24, 18, 14 (from left to right). Scale bars, 100 μm. (**D**) Light micrographs of adult wings bearing indicated genotypes are shown. Quantification of wing size (C’), n = 12, 11, 12, 11 (from left to right). Mean ± SD; n.s., not significant; ****P* < 0.001; two-tailed Student’s t-test ( A’, B’, and C’) or ordinary one-way ANOVA test (D’). Scale bars, 200 μm.

**
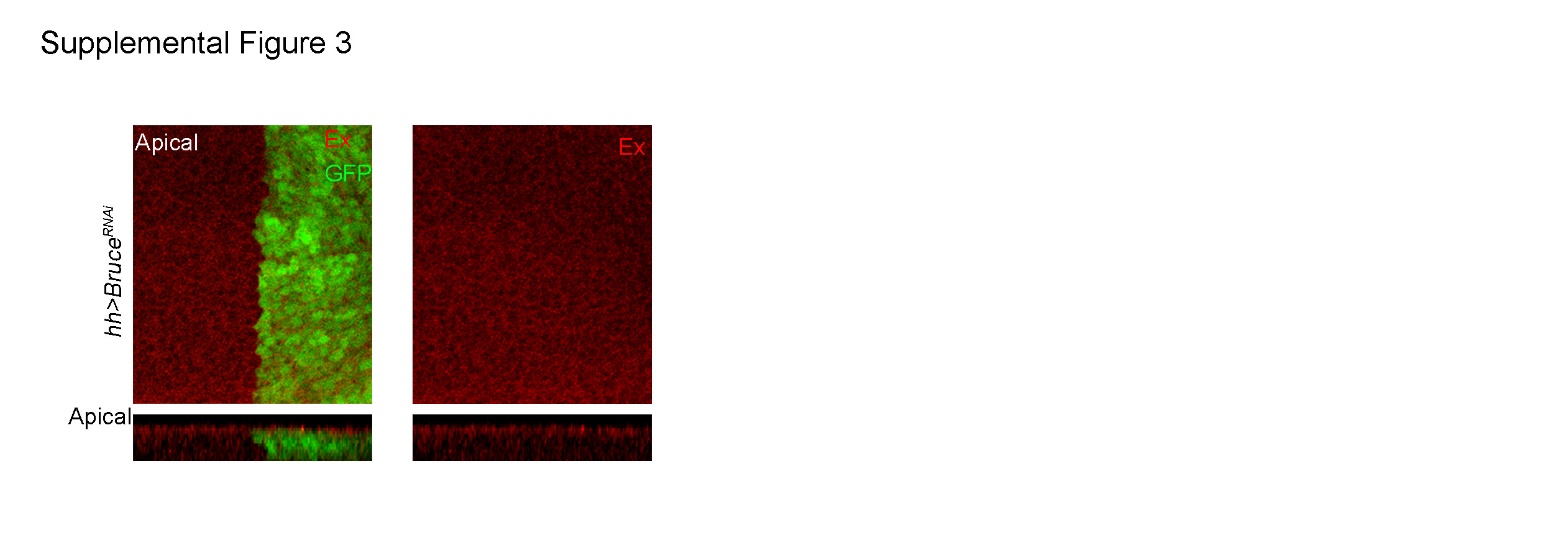
Supplementary Figure 3. Bruce depletion did not affect the localization of Ex.**

Wing pouch regions of *hh> Bruce^RNAi^* are stained with anti-Ex; apical, and cross-section views are shown.

**
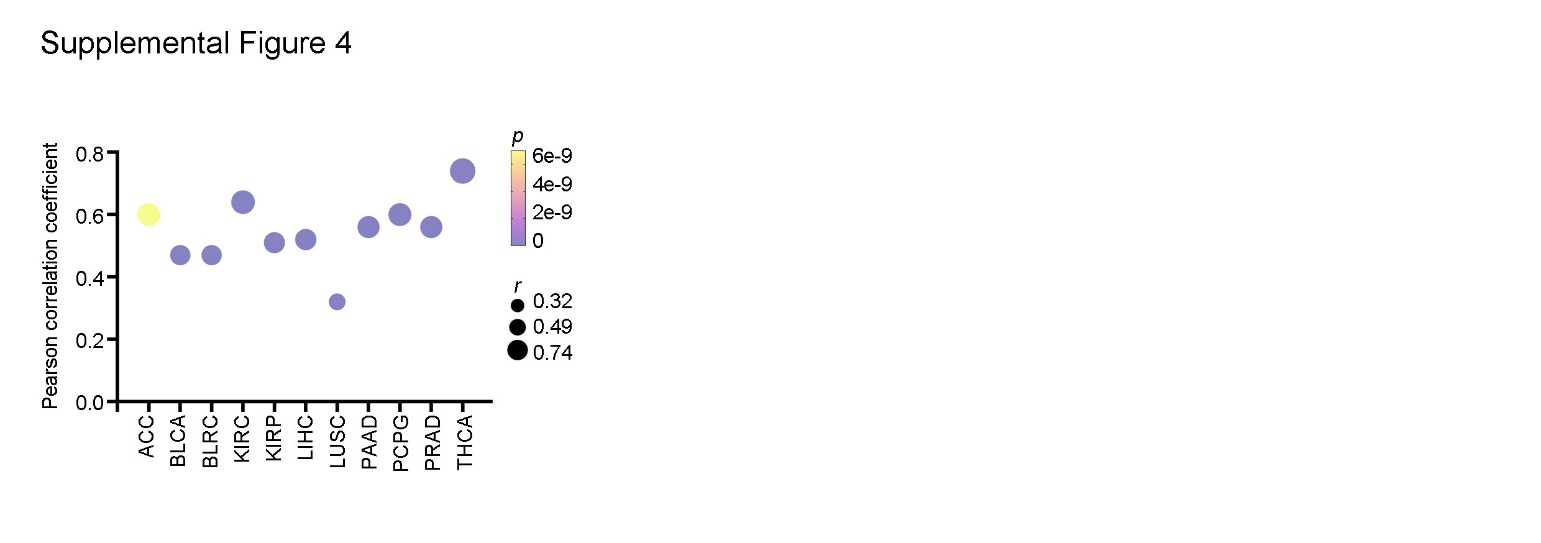
**

**Supplementary Figure 4. Bruce expression is positively correlated with SH3RF1 in multiple cancers.**

Heat map of the correlation coefficients between *Bruce* and *SH3RF1* in TCGA patients, which are displayed in different colors and size. The size scale indicates the degree of correlation coefficient. The color of the points is based on the p-value.
