# Supplementary figures and images for: E2 enzyme Bruce negatively regulates Hippo signaling through POSH-mediated expanded degradation
Source: Cell Death Dis. 2023 Sep 12;14(9):602. doi: 10.1038/s41419-023-06130-2 (PMC10497580; doi:10.1038/s41419-023-06130-2)

Figure 3B

WB: Myc


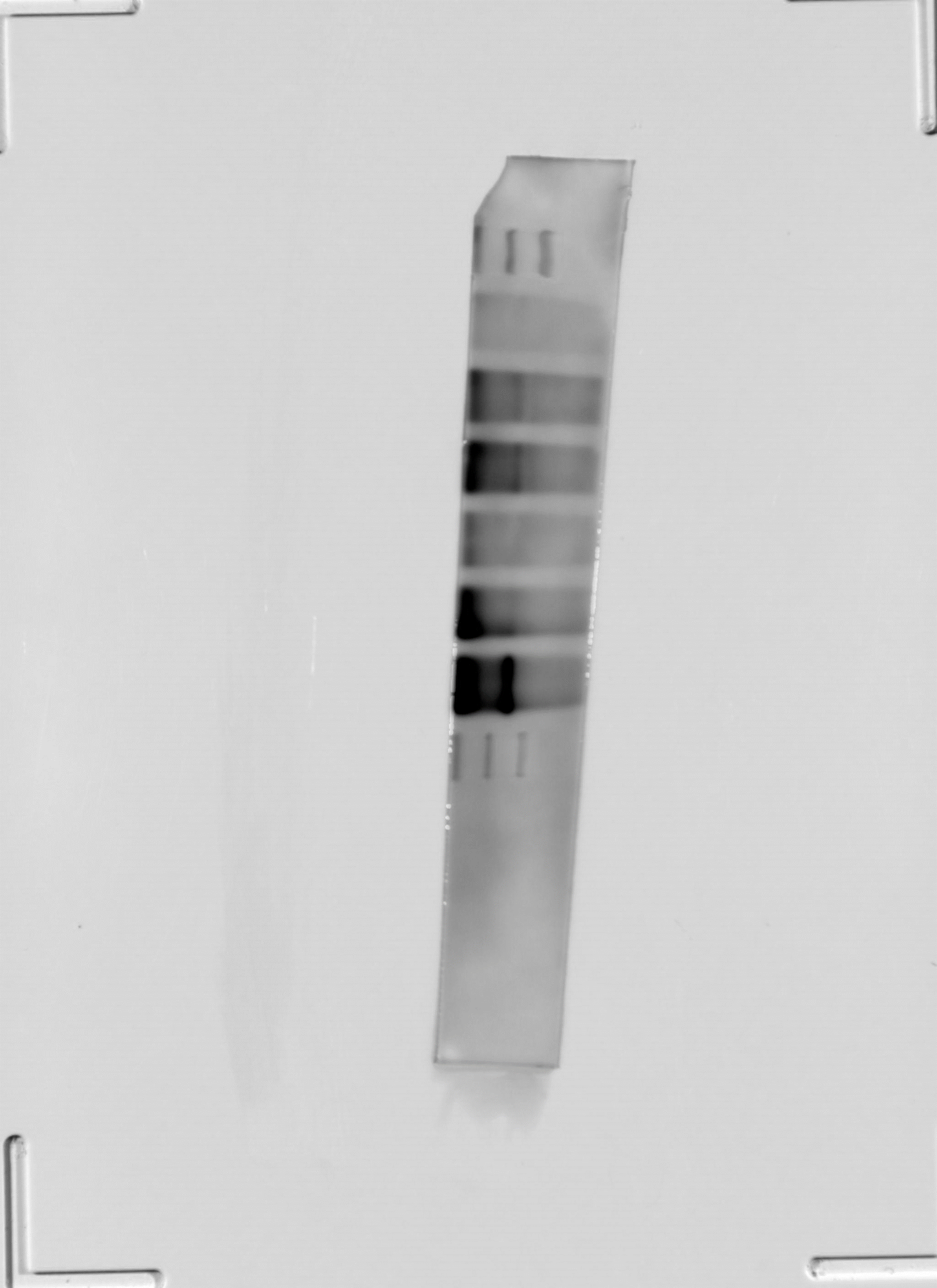


WB: HA


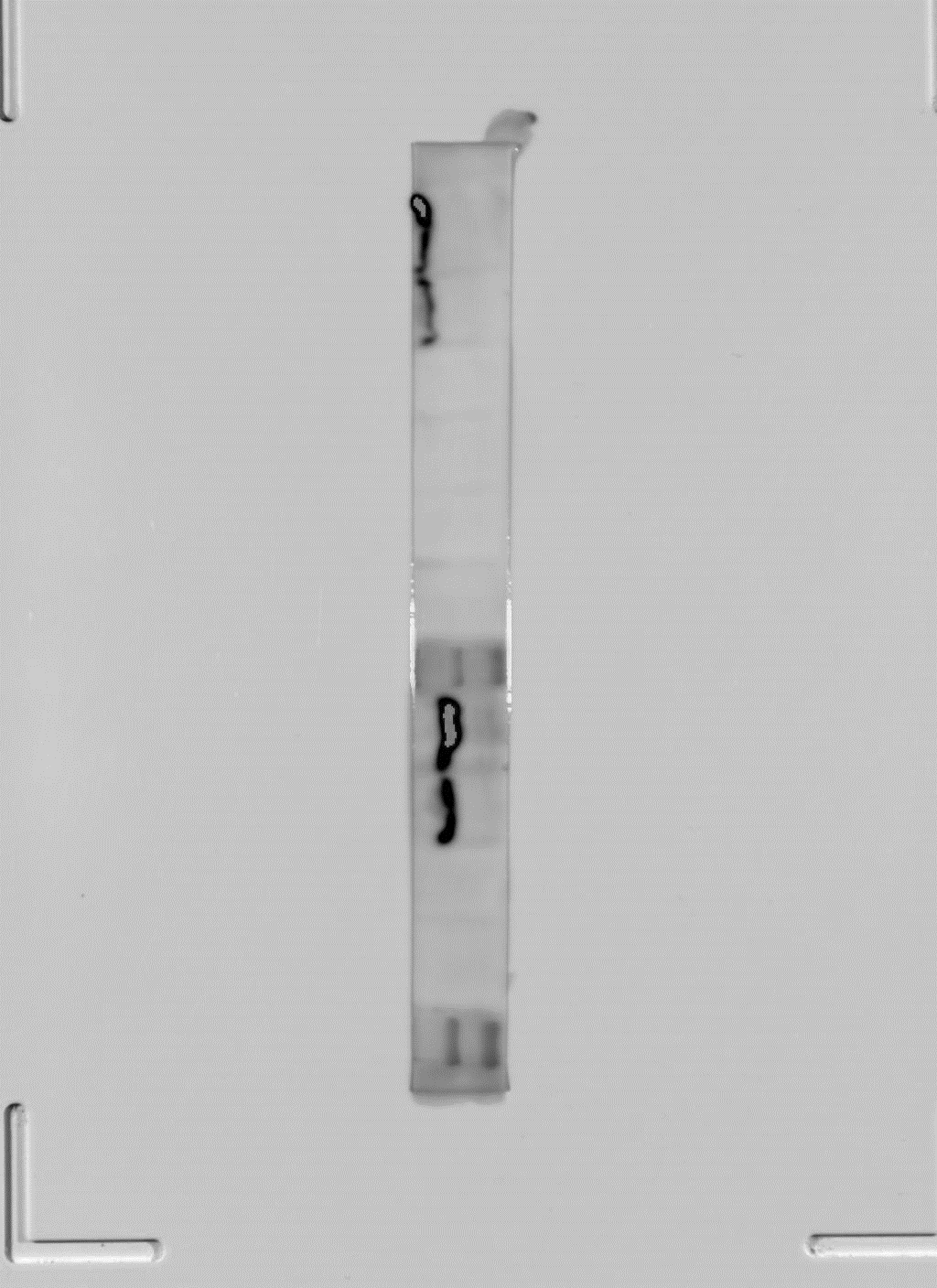


WB: b-actin


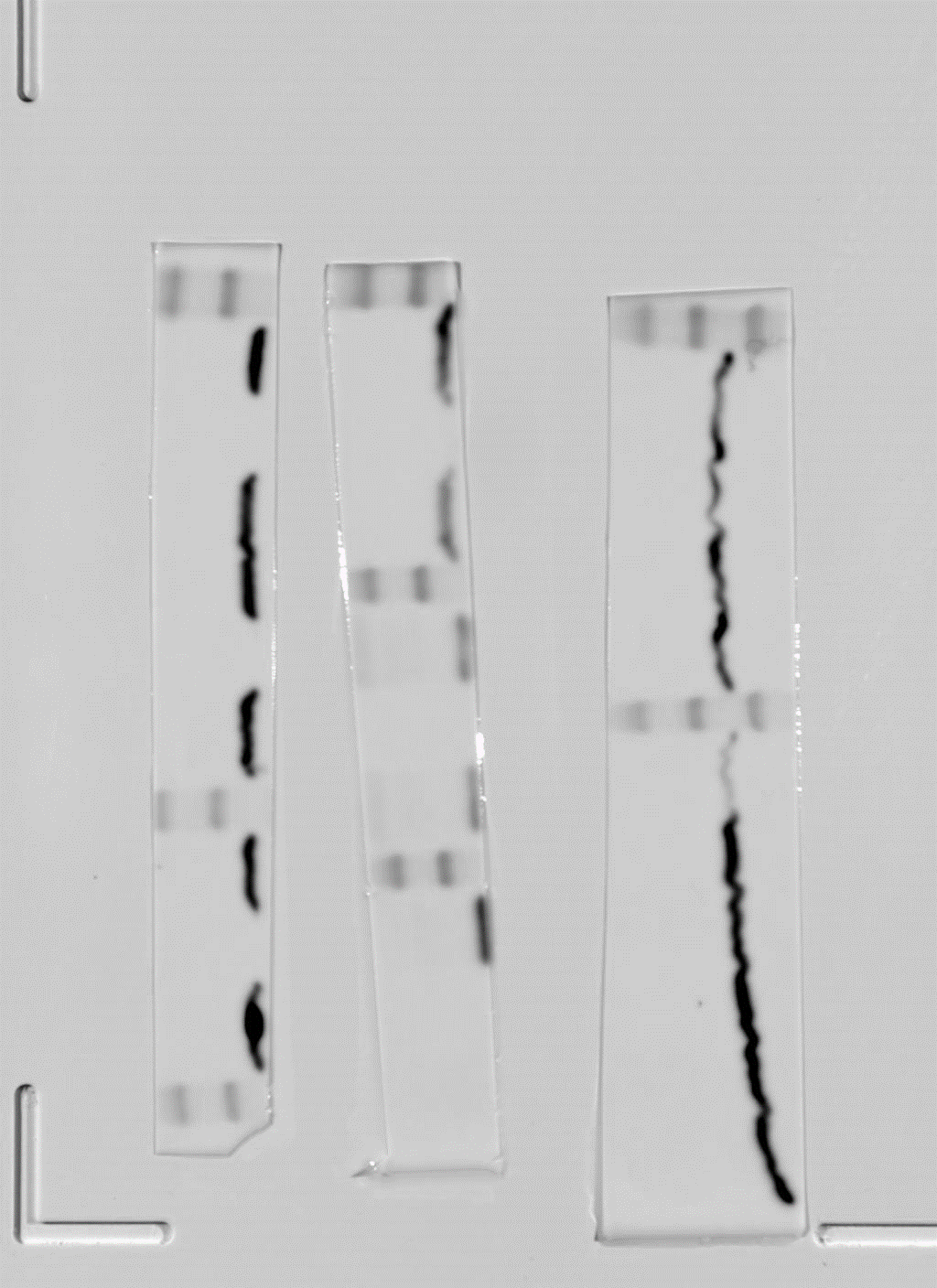


Figure 3C

IP: Myc

IB: Ubi


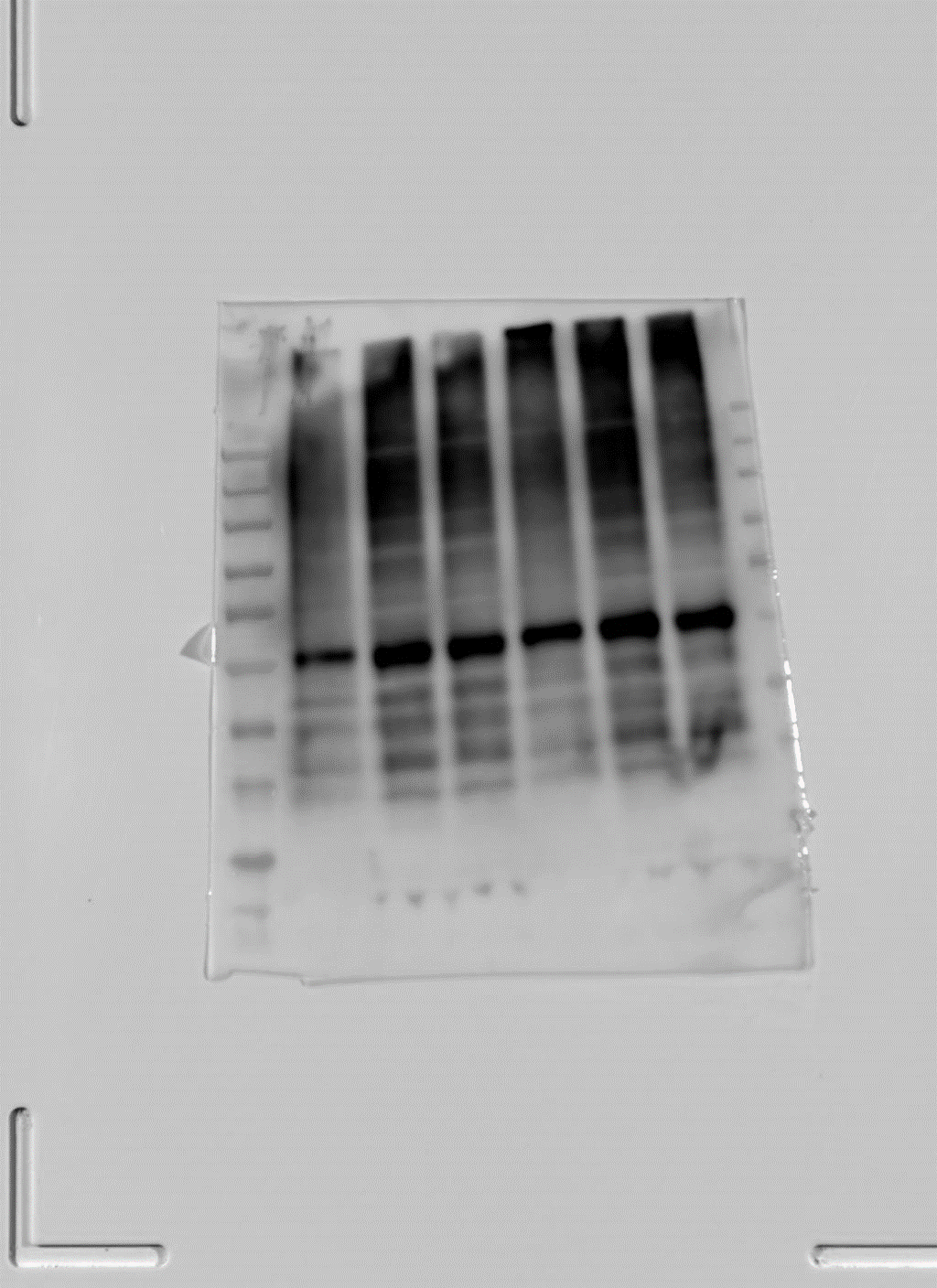


IB: HA


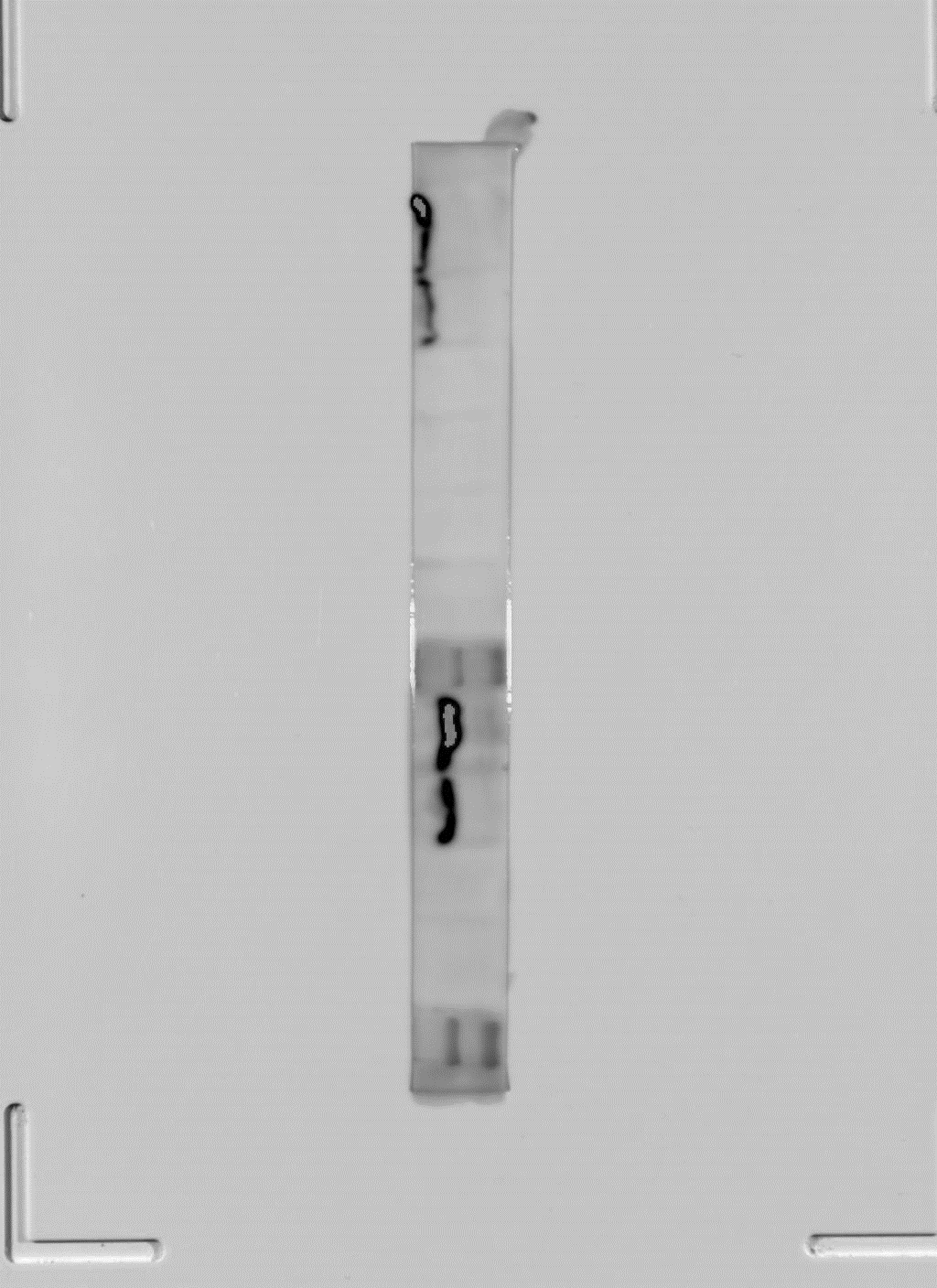


IB: Myc





IB: b-actin


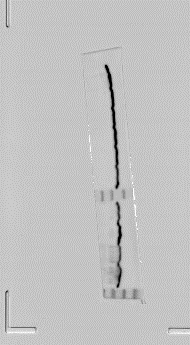

Supplement: Supplementary file 3 — original data files [file 41419_2023_6130_MOESM3_ESM.docx]
